# Surgical Treatment of Pseudoaneurysm of the Right Hepatic Artery Following Cholecystectomy: A Case Report

**DOI:** 10.7759/cureus.61858

**Published:** 2024-06-06

**Authors:** Abdellah Nouri, Ahmed Bensaad, Youssef Ghaddou, Sair Khalid, Fadil Abdelaziz

**Affiliations:** 1 Surgery, Mohammed VI University of Health Sciences, Cheikh Khalifa International University Hospital, Casablanca, MAR; 2 Surgical Gastroenterology, Mohammed VI University of Health Sciences, Cheikh Khalifa International University Hospital, Casablanca, MAR; 3 General Surgery, Mohammed VI University of Health Sciences, Cheikh Khalifa International University Hospital, Casablanca, MAR

**Keywords:** surgical ligation, complications, laparoscopic cholecystectomy, right hepatic artery, pseudoaneurysm

## Abstract

Pseudoaneurysms of the right hepatic artery following cholecystectomy are caused by either vascular damage or erosion after a biliary leak. Symptoms often include haemobilia, melena, vomiting, jaundice, and hemodynamic failure due to aneurysm rupture. The ideal treatment is arterial embolization or, in rare cases, stenting.

We present a case of pseudoaneurysm of the right hepatic artery post-laparoscopic cholecystectomy. The patient presented with abdominal pain, vomiting, and hemodynamic failure on postoperative day 45. Magnetic resonance imaging (MRI) showed a large hematoma and a pseudoaneurysm of the right hepatic artery. A laparotomy was performed, and a large hematoma was found and evacuated. After the pringle maneuver, the pseudoaneurysm was resected. The right hepatic artery was ligated with clips, and a sub-hepatic drain was placed.

The non-availability of emergency embolization forced surgical closure of the right hepatic artery, which is still the first-line treatment for such cases. Injury of the right hepatic artery is a rare complication, often overlooked by surgeons, and requires early diagnosis. Surgical treatment is reserved for cases of embolization failure or hemodynamic instability.

## Introduction

Cholelithiasis affects 10% to 15% of the Western population [1]. The standard treatment for symptomatic gallstones is laparoscopic cholecystectomy [1]. Biliary duct injury complicates 0.6% of cholecystectomies and is often associated with injury to the right hepatic artery, which manifests as a pseudoaneurysm. These patients may also present with haemobilia or hemoperitoneum [2].

Diagnosis is primarily based on angiography or magnetic resonance imaging (MRI). The ideal treatment is embolization, with surgical treatment involving ligation of the right hepatic artery reserved for cases when embolization fails or the patient is hemodynamically unstable. We present a case of pseudoaneurysm of the right hepatic artery following laparoscopic cholecystectomy, which was diagnosed by MRI, and treated by surgical ligation of the right hepatic artery.

## Case presentation

A 48-year-old patient had a laparoscopic cholecystectomy 45 days ago, followed by a second operation for biliary peritonitis after four days of cholecystectomy and peritoneal cleansing with drainage of the biliary tract using a T-tube drain. The patient was discharged on postoperative day (POD) 5 and his drain was removed four weeks later.

**Figure 1:**
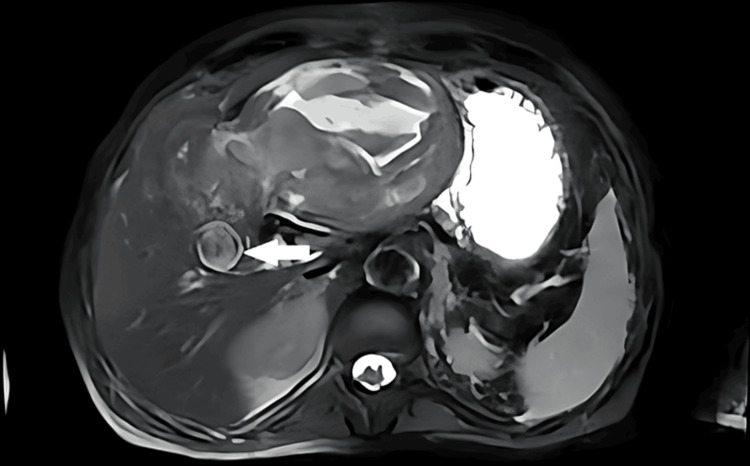
Magnetic resonance imaging showing pseudoaneurysm of the right hepatic artery (arrow) with large hematoma.

On the 45th day post cholecystectomy, the patient presented to our center with abdominal pain, vomiting, and hemodynamic instability. After hemodynamic resuscitation, the MRI was performed and showed a large hematoma associated with a pseudoaneurysm of the right hepatic artery (Figure 1).

Initial laboratory investigations portrayed a hemoglobin of 8 g/dL, hematocrit value of 20.3%, and platelet count of 310 × 10^3^ cells/µL (Table 1).

**Table 1:** Laboratory data for our patient.

Parameter	Normal laboratory value	Value
Hemoglobin g/dl	13-18	8
Hematocrit %	39-53	20.3
Platelet 10^3 ^/uL	150-400	310

**Figure 2:**
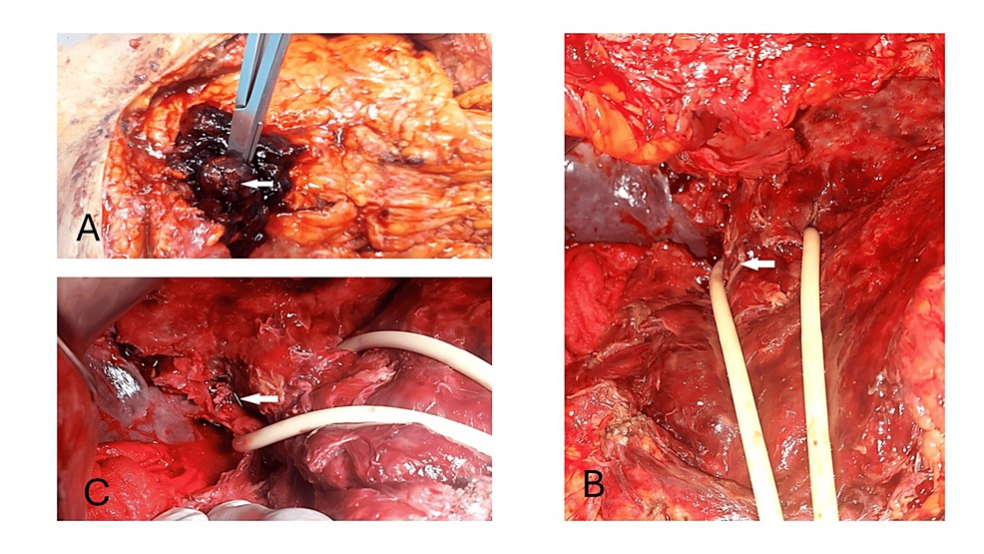
Per-operative findings. (A) Pseudoaneurysm (arrow) with large hematoma. (B) Injury of the right hepatic artery after dissection (arrow). (C) Ligation of the right hepatic artery with clips (arrow).

A subcostal laparotomy was performed, and a large hematoma was found and evacuated, after the pringle maneuver, the pseudoaneurysm was resected, then the right hepatic artery was ligated with clips. A subhepatic drain was placed (Figures 2A-2C).

The patient spent 14 days in intensive care following a pneumopathy treated with broad-spectrum antibiotics and was discharged on POD 20. The drain returned 30 cc of bile in the first week, 10 cc during the next 18 days, and was removed once the bile drainage stopped.

## Discussion

The right hepatic artery injury is a well-documented complication of cholecystectomy, it may be isolated in 7% of cases in an autopsy series of cholecystectomy patients or associated with an injury to the common bile duct in 20% to 40% of cases [3,4]. These vascular injuries are either iatrogenic or caused by erosion due to biliary leakage, which manifests itself as a pseudoaneurysm [4]. The time between cholecystectomy and clinical signs can range from six days to five years, with an average of 36 days [3,4]. Symptoms often include haemobilia (90%), melena, vomiting, jaundice due to compression of the bile duct caused by the hemoperitoneum [5,6], and shock often due to aneurysm rupture [7].

The ideal treatment for extrahepatic aneurysms is arterial embolization or, rarely, stenting to close the breach [2,8]. The advantage is easy access to the breach, which avoids the morbidity of surgery, and in the event of failure, the surgical excision of the aneurysm and ligation of the right branch of the hepatic artery are the most common procedures [8]. A preoperative study of anatomical variations and preoperative cholangiography can help prevent vascular lesions [8].

Our patient underwent surgery for biliary peritonitis on POD 4, following a biliary leak in which a T-tube drain was placed in the biliary tract and then removed four weeks later. On the 45th day post cholecystectomy, abdominal pain and vomiting prompted an MRI, which revealed a hematoma and a pseudoaneurysm of the right hepatic artery. Peritonitis or an intra-abdominal collection was suspected. This pseudoaneurysm was probably due to an unrecognized injury in the right hepatic artery or to erosion following the patient's biliary leak. Surgical ligation of the right hepatic artery was indicated due to the non-availability of emergency embolization and remains a first-line treatment in our situation.

## Conclusions

Pseudoaneurysms of the right hepatic artery following cholecystectomy due to either injury or erosion of this artery following a biliary leak. This is a rare complication often neglected by surgeons, manifesting as vomiting, hemoperitoneum, and haemobilia. The ideal treatment is embolization, with surgery advised in cases of hemodynamic instability or embolization failure. To prevent this complication, it is important to conduct radiological studies, perform open surgery if necessary, and adhere to safety protocols.
